# Placental nitrosative stress and in utero exposure to particulate matter

**DOI:** 10.1186/2049-3258-73-S1-P17

**Published:** 2015-09-17

**Authors:** Nelly Saenen, Karen Vrijens, Bram Janssen, Narjes Madhloum, Martien Peusens, Wilfried Gyselaers, Charlotte Vanpoucke, Wouter Lefebvre, Harry Roels, Tim Nawrot

**Affiliations:** 1 Hasselt University, Diepenbeek, Belgium; 2 Department of Obstetrics, East-Limburg Hospital, Genk, Belgium; 3 Belgian Interregional Environment Agency, Brussles, Belgium; 4 Flemish Institute for Technological Research (VITO), Mol, Belgium

## Background and aims

A wide variety of adverse health effects on both fetuses and neonates have been ascribed to particulate matter (PM) air pollution. Recent evidence suggests that PM exposure results in increased oxidative and nitrosative stress. In the ENVIR*ON*AGE birth cohort, we investigated the association of placental 3-nitrotyrosine (3-NT) with PM exposure during various time windows of pregnancy.

## Methods

3-NT levels were measured in 341 placental tissue samples, selected from the ENVIR*ON*AGE birth cohort, using a competitive ELISA. Daily PM_10_ and PM_2.5_ exposure levels were interpolated for each mother's residential address using a spatiotemporal interpolation method in combination with a dispersion model. Multiple linear regression models were used to assess the association between 3-NT and PM exposure for different pregnancy windows.

## Results

The placental 3-NT level, adjusted for gender, gestational age, maternal age, pre-gestational BMI, smoking status, and warm or cold period at delivery, raised with 31.0% (*p* = 0.0008) for an interquartile range increment in whole pregnancy PM_2.5_ exposure. The association was driven by PM_2.5_ exposure during the first trimester (25.7 %, *p* = 0.01) and second trimester (37.0%, *p* = 0.003) of pregnancy.

## Conclusions

We observed a positive association between 3-NT levels in the placenta and PM_2.5_ exposure during whole pregnancy. Our findings, which are in line with experimental evidence on cigarette smoke and diesel exhaust exposure, indicate the influence of PM exposure during pregnancy on placental oxidative stress. The impact of placental 3-NT with regard to PM exposure on newborn's health needs further elucidation.

